# Components of Oral Health Related to Motor Impairment in Children With Neuropsychiatric Disorders

**DOI:** 10.7759/cureus.46093

**Published:** 2023-09-27

**Authors:** Desislava A Konstantinova, Lyubomir G Dimitorov, Ana N Angelova, Rouzha Z Pancheva

**Affiliations:** 1 Department of Dental Material Science and Prosthetic Dental Medicine, Medical University “Prof. Dr. Paraskev Stoyanov”, Varna, BGR; 2 Department of Neurosurgery, Medical University “Prof. Dr. Paraskev Stoyanov”, Varna, BGR; 3 Department of Dentistry, Medical University “Prof. Dr. Paraskev Stoyanov”, Varna, BGR; 4 Department of Hygiene and Epidemiology, Medical University “Prof. Dr. Paraskev Stoyanov”, Varna, BGR

**Keywords:** pediatric, oral mucosa, physical disability, oral status, neurological impairment

## Abstract

Introduction

Oral health significantly influences functions such as chewing, swallowing, and overall well-being. Children with neuropsychiatric disorders (NPD) often experience compromised oral functions, escalating their risk of malnutrition.

Materials and methods

Our study, conducted in Varna, Bulgaria, from April to October 2017, aimed to evaluate some components of the oral health of 49 children with NPD and its relation to their motor impairments. In the studied cohort, participants were categorized based on their Gross Motor Function Classification System (GMFCS) scores into two groups: minor limitations (ML), encompassing GMFCS levels 1-3, and gross limitations (GL), which included GMFCS levels 4-5. Comprehensive oral examinations were conducted by a trained dentist. Data analysis utilized the JAMOVI v.2.2.2.0 software with a 0.05 significance threshold.

Results

Preliminary findings indicate that children with more pronounced motor limitations have poorer oral health compared to their mildly impaired counterparts. A mere 14.3% (n=7) of the children with NPD had recorded dental visits. The data show that 18.2% (n=6) of ML children had at least an annual dental consultation, while only a single child (6.2%) from the GL group had a dental visit, leaving a staggering 93.8% (n=15) without any.

Statistical analyses indicate a significant relationship between motor activity (MA) and toothbrushing frequency (r=0.529, p=0.0001), suggesting that children with better MA have improved chances of maintaining oral hygiene. A significant correlation was observed between dental visits and toothbrushing frequency (r=0.371, p=0.0007).

Conclusion

Given their challenges, children with NPD require increased attention to dental care, emphasizing regular checkups and preventive oral health measures. This study prompts a reevaluation of these care standards.

## Introduction

The oral cavity has several main functions, such as chewing, swallowing, and communication [1]. Therefore, oral health is a key aspect of health, life satisfaction, quality of life, and self-perception [2]. Impairment of oral function is common in children with neuropsychiatric disorders (NPD), and this unfavorable feature may indirectly interact with functional deterioration of oral health (poor personal oral hygiene (POH), many pathological processes, and/or inadequate dental restorations), leading to a high risk of malnutrition. These children have specific care needs and exhibit a variety of oral symptoms, daily living issues, and worries about a kid’s oral health and its effects on both the child and the family’s quality of life [3]. There are a small number of studies that report the periodontal health status of children with neurodevelopmental disorders. The majority of studies focus mainly on dental caries [4-6]. A factor in the ambiguous interpretation of the facts are the many indices used to measure periodontal health.

Children and adolescents with autism spectrum disorders (ASD) have poorer oral hygiene and a higher rate of periodontal disease than non-autistic individuals [7]. Poor oral hygiene is considered to be the leading cause of gum disease. The World Health Organization (WHO) has predicted a prevalence rate of ASD at 1:160 people at the global level [8]. People with ASD need help and easier access to dental professionals [9,10]. Training dental teams and informing parents would help ensure a higher standard of dental treatment. The aim of our study is to assess some components of the oral health of children with NPD and to study the relationship of pathological processes in the oral mucosa with their motor impairment.

## Materials and methods

Study setting and participants

The study was conducted in Varna, spanning seven months, from April to October 2017. It was approved by the Ethics Committee of Research at the Medical University of Varna with Protocol Number 60/23.2.2017. A total of 49 children with neuropsychiatric diseases hailing from Varna and Ruse were included. Among these, children were either housed in residential care homes (n=24) or lived in a family environment (n=25). The breakdown of the children based on their diagnosis was as follows: 23 children with cerebral palsy (CP), four with hydrocephalus, five with mild mental retardation, seven with Down syndrome and other syndromes, and 10 with autism.

Examination procedure

All children were examined under alternative clinical conditions, in an environment familiar to them. Those who exhibited active independent movements were seated in an office chair suitable for their condition, equipped with a headrest. Meanwhile, those who used their wheelchairs for mobility were examined without relocation. Directed lighting was provided via a mobile artificial light source. The examinations utilized a 2.5× magnification and additional directive lighting. Disposable dental examination kits were used. The results from the examination were recorded on a specially designed diagnostic card. General patient data, including age, gender, primary disease, and other relevant information, were collected in the initial phase of the examination.

Extraoral examination

Before proceeding with the extra- and intraoral examinations of the children with neurological functional problems (NFP), explanations about the examination methodology were given, and informed consent was duly obtained from their parents or guardians. The extraoral examination entailed checking the head for symmetry in relation to the sagittal and transverse planes, spotting visible scars on the face and neck, identifying skin lesions, and observing other pathological changes that might be linked to the functional adequacy of the masticatory apparatus.

Intraoral examination

The intraoral examination encompassed a comprehensive dental review. This involved determining the dental status and noting findings using globally accepted symbols with corresponding indications.

Decayed, Missing, and Filled Index (DMF/dmf Index)

The Decayed, Missing, and Filled Index (DMF for permanent teeth and dmf for deciduous or milk teeth) can be utilized with the number of affected teeth as the numerator (dmft) or the number of affected surfaces (dmfs). The mean DMFT is used as the standard to compare dental health in many publications, including the World Health Organization (WHO) Oral Health Databank. Using it to measure caries increments in individuals over time is complex unless detailed treatment records are available for the interim period. Thus, the DMF is most valuable for young people, but the data must be age-specific, typically avoiding ages between six and 12 years due to mixed dentition.

Clinical evaluation of oral hygiene status

The Simplified Oral Hygiene Index (OHI-S) using light illumination, a mouth mirror, a diagnostic probe, and a standard WHO periodontal probe was used.

Oral mucosal examination

The characteristics of the oral mucosa were documented based on the following classification of etiology: traumatic injuries, infectious diseases, aphthous changes, allergic and toxic alterations, changes in some systemic diseases, changes due to specific infections, stomatitis, tongue anomalies and diseases, lip diseases, precancerous conditions of the oral mucosa, and neoplasms of the oral mucosa. Diseases of the oral mucosa were identified based on their localization as stomatitis, gingivitis, papillitis, glossitis, and cheilitis.

For many of these conditions, specific classification schemes were developed. Based on their pathogenesis, they were categorized as either acute or chronic. They were further divided into primary diseases (those caused by local factors) and secondary diseases (those that accompany another primary disease, known as symptomatic or satellite stomatitis).

Dental health habit assessment

Apart from the instrumental examination, basic information regarding children’s dental health maintenance habits was collected. This encompassed questions about teeth brushing routines, including who conducts the brushing, the frequency of brushing daily, and the regularity of visits to a dental specialist.

Statistical analysis

Summaries including means and standard deviations, and absolute and relative frequencies were used to describe the study sample. Chi-square tests compared associations between categorical variables, whereas an independent samples t-test was used to test for differences in continuous variable outcomes between children living in residential care and family environment. Statistical data processing was performed using the statistical package JAMOVI v.2.2.2.0 at a 0.05 level of significance.

## Results

In the studied cohort, participants were categorized based on their Gross Motor Function Classification System (GMFCS) scores into two groups: minor limitations (ML), encompassing GMFCS levels 1-3, and gross limitations (GL), which included GMFCS levels 4-5. The age distribution between ML and GL children was similar, and there was an approximately even gender distribution across both groups. Half of the participants resided in institutional care facilities, while the other half lived with their families. Ethnically, a majority of ML children were of Bulgarian origin, while most GL children were Roma. Further demographic details can be found in Table 1.

**Table 1:** Basic characteristics of the sample Significant differences are marked with *. IQR: interquartile range

Characteristics	Minor limitations (n=33)	Gross limitations (n=16)	p-value	U/Fisher’s test
Age (years) (median±IQR)	3.92±2.83	4.46±3.67	0.856	255
Male (%)	45.5%	56.3%	0.551	0.503
Male (number)	15	9	N/A	N/A
Gestational age at birth, (weeks) (median±IQR)	38±8.5	34±6	0.041	127*
Growing environment	N/A	N/A	0.016	6.44*
Residential care (%)	36.4%	75%	N/A	N/A
Family environment (%)	63.6%	25%	N/A	N/A
Ethnicity	N/A	N/A	0.008	9.12*
Bulgarian (%)	75.8%	31.3%	N/A	N/A
Turkish (%)	9.1%	31.3%	N/A	N/A
Roma (%)	15.2%	37.5%	N/A	N/A
Diagnosis	N/A	N/A	<0.001	15.7*
Cerebral palsy (%)	27.3%	87.5%	N/A	N/A
Mixed diagnosis (%)	72.7%	12.5%	N/A	N/A

Dental visits

A mere 14.3% (n=7) of the children with NPD had recorded dental visits. Specifically, 18.2% (n=6) of ML children had at least an annual dental consultation, while only a single child (6.2%) from the GL group had a dental visit, leaving a staggering 93.8% (n=15) without any.

Toothbrushing habits

**Figure 1:**
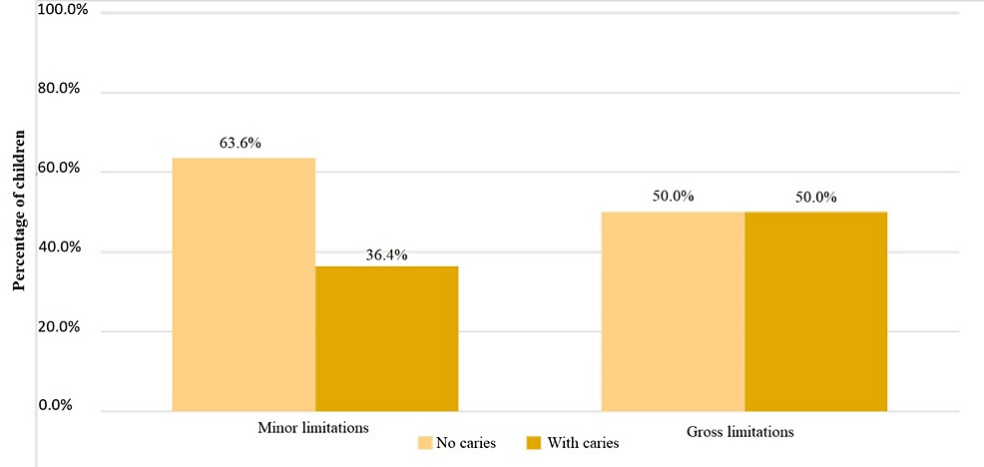
Percentage of children with caries by type of motor limitation

It was alarming to note that all the children in the GL category did not engage in any personal oral hygiene (POH), signifying none brushed their teeth daily. This neglect poses potential risks for oral diseases such as caries, periodontal inflammation, and associated complications. Conversely, ML children displayed better oral hygiene habits, but still, less than half engaged in the recommended twice-daily toothbrushing. Figure 1 offers a detailed representation.

Statistical analyses indicate a significant relationship between motor activity (MA) and toothbrushing frequency (r=0.529, p=0.0001), suggesting that children with better MA have improved chances of maintaining oral hygiene. Additionally, a significant correlation was observed between dental visits and toothbrushing frequency (r=0.371, p=0.0007). Regular dental consultations seem to bolster the motivation for children to maintain consistent oral hygiene practices.

DMF Index

**Figure 2:**
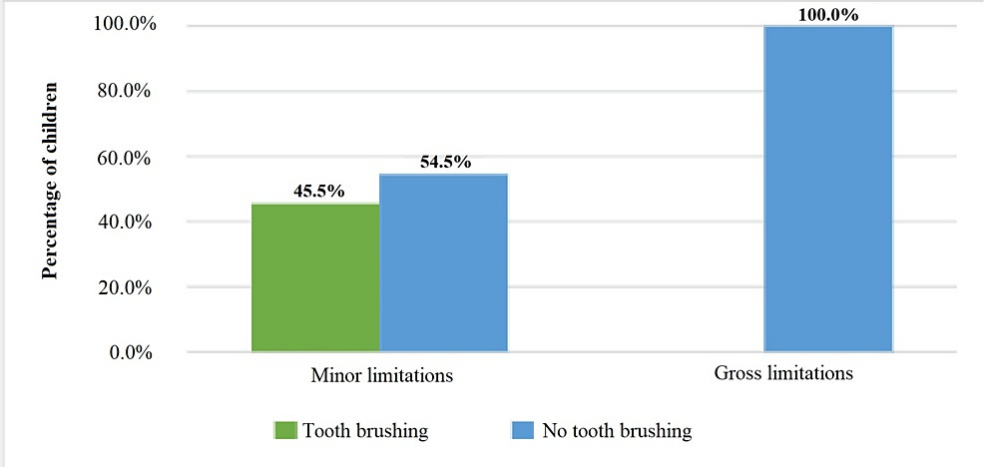
Percentage of children practicing toothbrushing by motor limitation type

**Figure 3:**
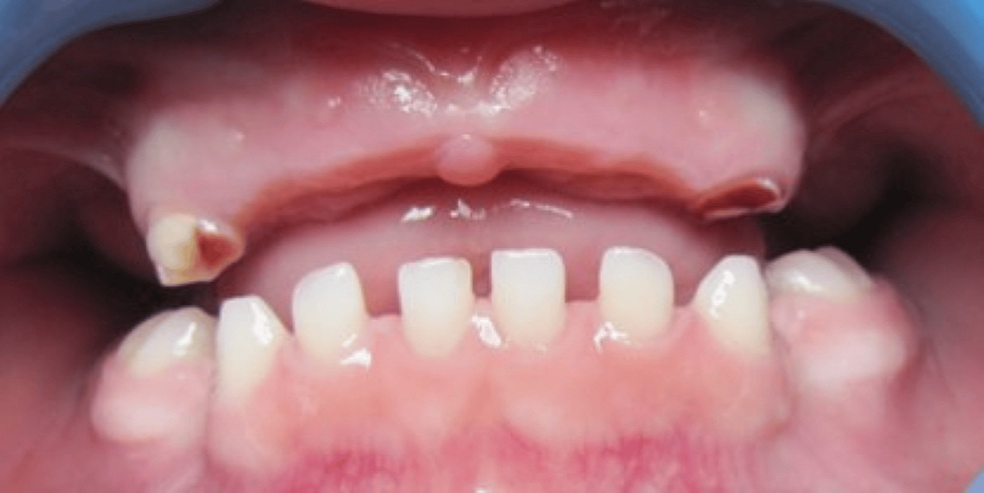
Intraoral view of a boy with gross limitations

**Figure 4:**
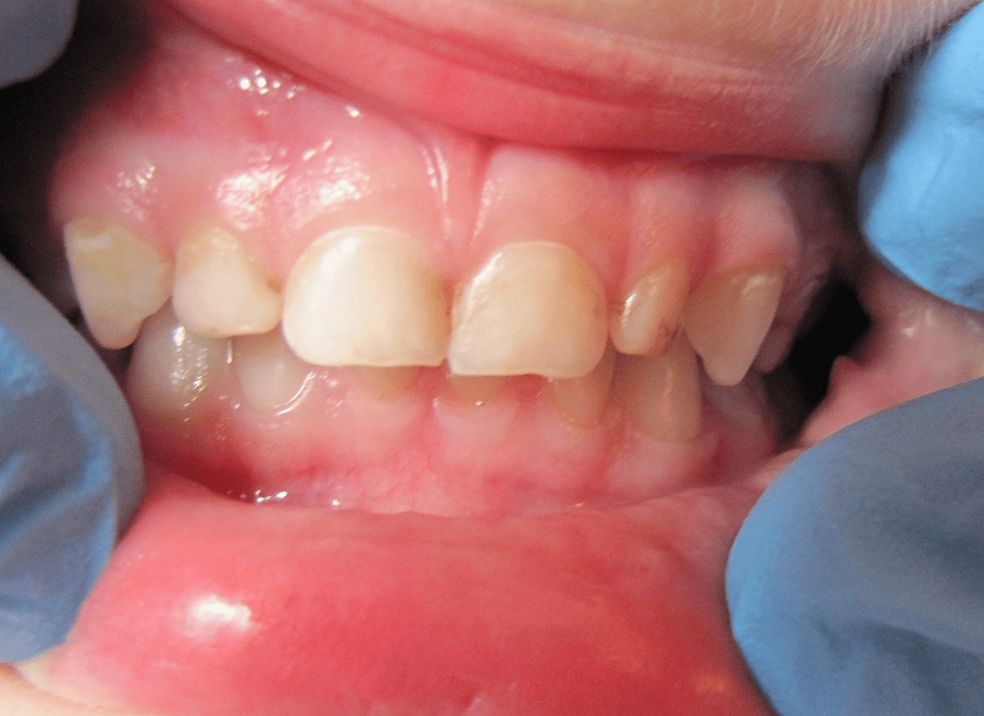
Intraoral view of a girl with minor limitations

A contingency table assessed the relationship between the Gross Motor Function Classification System (GMFCS) and the presence of dental caries. For children with minor limitations, 63.6% (n=20) showed no caries, while 36.4% (n=13) had caries. In contrast, children with gross limitations had an even split, with 50% displaying no caries and the remaining 50% with caries. Out of the 49 children examined, 59.2% (n=29) were caries-free, and 40.8% (n=20) exhibited caries. Statistical testing using the chi-square (χ²) method yielded a value of 0.829 with a p-value of 0.362, suggesting no significant association between GMFCS levels and the prevalence of caries in the sample studied (Figures 2-4).

Oral mucosa

A variety of pathogenic agents and damaging factors can cause various functional and structural disorders of the oral mucosa. These disorders present with predominant pathological reactions or typical pathological processes characteristic of the respective disease. The main classical clinical manifestations of inflammation are local and general, described in antiquity, and expressed by redness, edema, pain, and impaired function. The causes of inflammation can be biological (microorganisms and their toxins, viruses, fungi, and other parasites, plant and animal poisons, allergic reactions, etc.), physical (traumatic), chemical, and physicochemical pathogens.

**Figure 5:**
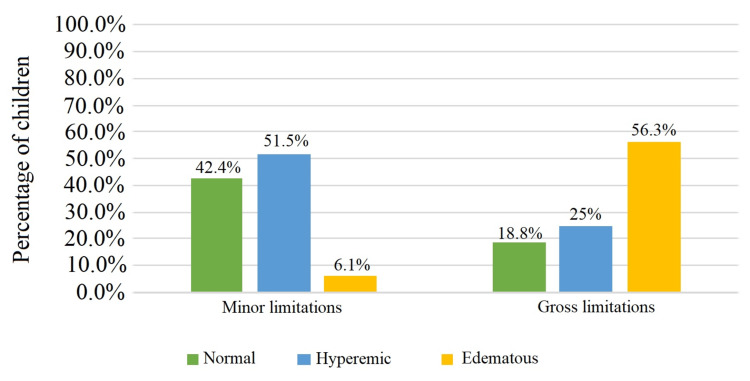
Percentage of children with pathological oral mucosal changes by type of motor limitations

**Figure 6:**
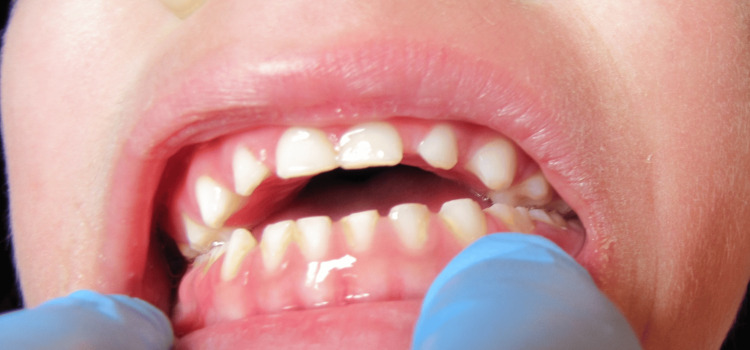
Pathological oral mucosal changes in the area of lower molars

The oral mucosa of our ML and GL patients was separated into three groups: normal, hyperemic, and edematous. The findings of the oral mucosa examination indicate that more than 90% of children with ML have normal or hyperemic oral mucosa, whereas among the GL patients, predominant is the edematous oral mucosa (more than half of the children are with this type of mucosa). Statistical analysis solidified these observations, confirming a significant association with a χ² value of 15.6 (p<0.001) (Figures 5, 6).

Inflammatory and traumatic lesions of masticatory mucosa

**Figure 7:**
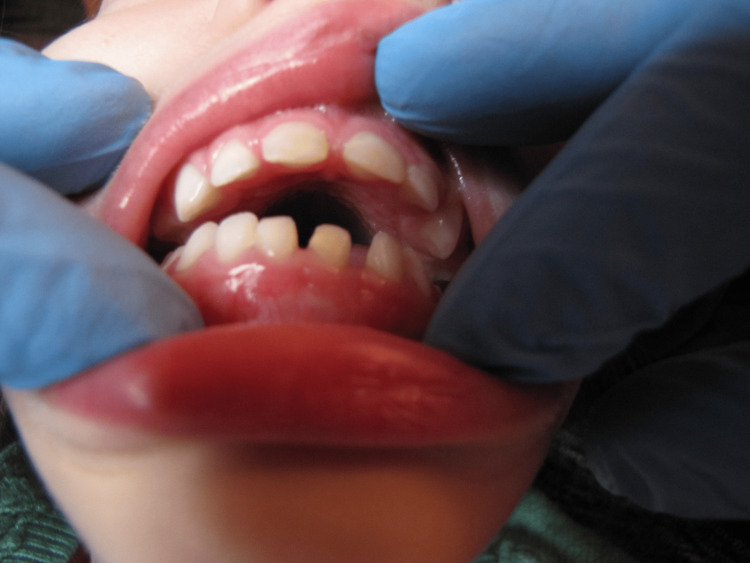
Intraoral view of a boy with gingivitis

The results of the study of both groups of children with neurodevelopmental diseases show that aphthous changes are the most common in all studied children. Based on the localization, diseases of the oral mucosa are divided into stomatitis (in diffuse spread of the process) (10 children), gingivitis (in localization on the gums) (nine children), papillitis (affecting only papillae) (one child), cheilitis (diseases of the lips (10) and palate (five children)), and glossitis (diseases of the tongue) (0 children). A total of 18 children (34% of all) have normal mucosal coverage of the oral cavity (Figure 7).

## Discussion

This study provided basic information about oral health knowledge and practice in children with neuropsychiatric disorders. Children with NPD exhibit a heightened susceptibility to oral health issues, which can further complicate feeding difficulties [11]. Oral mucosa disorders can be a barrier to adequate nutritional intake. The World Health Organization advises the first dental checkup to be at six months, with subsequent checkups every six months unless there are exceptional circumstances [12-14]. However, our study revealed that a mere 13.5% of the NPD children surveyed had a dental visit. Such a lack of professional dental care implies that most have not benefited from routine oral cleaning and preventative care, potentially jeopardizing their oral health and related systemic health. The care of special needs children by parents in South Bedfordshire was hindered by a number of factors, according to Lee’s study on oral health views. It was found that 47% of parents undervalued the significance of oral health and that there was a corresponding lack of education about dental and oral health [15].

Effective personal oral hygiene (POH) primarily involves eliminating plaque and leftover food particles, with daily toothbrushing serving as a critical indicator. Alarmingly, our study identified that over 70% of participants neglect this basic oral care practice, leaving them vulnerable to oral complications. A notable correlation was found between dentist visit frequency and toothbrushing habits, underscoring the importance of regular dental checkups in instilling and reinforcing good oral care practices.

Delving deeper, Alshatrat et al.’s work on the Jordanian autistic population unveiled that individuals with ASD were noticeably less informed on oral health aspects, with fewer practicing daily brushing and only 15% capable of doing so independently [16]. Magoo et al. performed a study on parents of children suffering from autism spectrum disorders [17]. Study results showed that 71.2% were aware of the fact that primary teeth are important for good permanent dentition, while 61.5% refused any treatment for primary teeth. Once-a-day toothbrushing was performed by parents on their children in 82.7% of cases. These investigators also observed that parental knowledge regarding practical knowledge was restricted to only 39.37% of the study population. Further, disparities in the use of fluoridated toothpaste and mouth rinses between ASD and control groups were statistically significant. Our research further broadened this scope to highlight similar concerns in children with other conditions, such as cerebral palsy, hydrocephalus, mild cognitive impairment, Down syndrome, and other genetic anomalies. These children’s oral health status was intrinsically linked to their overall growth and nutritional well-being.

Echoing prior research, children with NPD inherently face greater oral health challenges than their healthier counterparts, making them more susceptible to subpar oral hygiene, elevated caries rates, and frequent mucosal issues [18-20]. In contrast, Glassman posited that while children and youths with unique health requirements did engage more in POH services, their oral health outcomes were paradoxically poorer than those without such needs [21]. This divergence from our findings underscores the multifaceted nature of oral health in special needs populations and calls for more nuanced understanding and tailored approaches.

Mucosal lesions can disrupt the chewing process in children, especially when they consume irritants such as certain foods and beverages. Our analysis revealed a significant incidence of mucosal pathology, affecting 20.80% of the study participants. A noteworthy correlation emerged between the regularity of toothbrushing in children with NPD, which dictates their ability to uphold optimal oral hygiene, and their dental visits. Regular dental consultations seem to bolster the commitment to personal oral hygiene (POH).

Autistic patients constitute a percentage of the special child population, and they require unique management because of their behavioral characteristics. Oral checkups and treatments are a challenge because of the reduced ability of autistic kids to communicate and manage their emotions, repetitive body movements, and hyperactivity associated with attention deficiency [22]. Prakash et al. confirmed that autism is a neurodevelopmental disorder that mainly affects the cognitive functions and patterns dependent upon these [23].

However, our study is not without its limitations. Oral health assessments conducted outside a clinic may not attain the precision of those done in fully equipped clinical environments. In our case, the absence of compressed air to dry teeth during assessments could have reduced our accuracy in spotting early lesions. Moreover, the diverse nature of the NPD children studied might restrict the general applicability of our findings.

Yet, as far as we are aware, this is the pioneering effort zeroing in on the oral health of children with NPD, especially in relation to motor impairments. The collaborative effort of a multidisciplinary team in this intricate study amplifies its value.

## Conclusions

Oral health in children with NPD remains a largely uncharted domain, often overshadowed by the primary ailment. Our findings spotlight prevalent oral mucosal disorders that can inflict pain and, if unaddressed, might lead to severe complications, including malnutrition. Infrequent dental checkups combined with subpar POH only amplify these oral challenges. Our data suggests that children with milder motor constraints fare better orally than their severely impaired counterparts. This research underscores the imperative to revisit and perhaps bolster dental care regimens for NPD children, given their unique challenges. This information will help in designing oral health education programs for these patients to improve their knowledge and motivate their families to practice preventive measures.
